# A pilot trial on safety and efficacy of erythrocyte-mediated steroid treatment in CF patients

**DOI:** 10.1186/1471-2431-6-17

**Published:** 2006-05-24

**Authors:** V Lucidi, AE Tozzi, S Bella, A Turchetta

**Affiliations:** 1 Ospedale Bambino Gesù, Department of Pediatrics, Cystic Fibrosis Unit, Rome, Italy; 2 Ospedale Bambino Gesù, Epidemiology Unit, Rome, Italy; 3 Ospedale Bambino Gesù, Department of Pediatrics, Respiratory Fisiopathology Unit, Rome, Italy

## Abstract

**Background:**

Chronic neutrophil inflammation of the respiratory tract tissues plays a key role in the pathogenesis and in prognosis of cystic fibrosis (CF). It is evident that an anti-inflammatory therapy represents an important step in the treatment of CF patients. Corticosteroids and ibuprofen have been proven to slow down the impairment of the pulmonary function in CF patients but their use is limited by the frequency of adverse events. A novel strategy for delivering low doses of steroids for long periods through the infusion of autologous erythrocytes loaded with dexamethasone has been recently set up. A recent study suggested the feasibility of therapy with low doses of corticosteroids delivered through engineered erythrocytes in CF patients. This study presents a further analysis of safety and efficacy of this therapy.

**Methods:**

The treatment group was not randomised and the assignment was based on the patient's consent. Patients entered the study if they had a forced expiratory volume in 1 second (FEV1) <70%, puberty development completed, pancreatic insufficiency, and chronic pulmonary infection requiring frequent cycles of intravenous antibiotic therapy. Patients were excluded if they underwent systemic corticosteriod therapy in the three months preceding the experimental treatment or were on therapy with non-steroidal anti inflammatory drugs (NASDs), or if they had liver CF disease, allergic bronchopulmonary aspergillosis, or positive tuberculin test. Controls were patients who followed a standard treatment, who fulfilled the enrolment criteria, and who were matched to the experimental group by gender, age, and severity of the disease.

**Results:**

Nine patients in the experimental group received the treatment once a month for a period of 24 month. Patients did not develop diabetes, cataract, or hypertension, or other typical side effects of steroid treatment during the follow up period. There was a constant improvement of FEV1 in patients undergoing the experimental treatment compared to a gradual decrease of the same parameter in the standard therapy group (P = 0.04). The average of clinic and radiological indexes did not vary. The number of infective relapses that have required antibiotic intravenous therapy was not different in the two groups, although the average of these episodes was slightly higher in the experimental therapy group.

**Conclusion:**

Intraerythrocyte corticosteroid treatment may stabilize the respiratory function in CF patients but is often considered too invasive by patients. The results obtained by our study may help planning an experimental, controlled, randomised study. A sample size of 150 patients per group would be sufficient for demonstrating such a difference with a 95% confidence interval and a power of 90%.

## Background

Chronic neutrophil inflammation of the respiratory tract tissues plays a key role in the pathogenesis of cystic fybrosis (CF). It has been shown that CF breast-fed children have a bronchopulmonary inflammation before the appearance of bacterial infections [1-3]. It is therefore evident that an anti-inflammatory therapy represents an important step in the treatment of CF patients [4].

Some authors already expressed the hope that uncertainties on the use of anti-inflammatory therapy in CF patients should have been removed [5], and new evidences supporting the role of inflammation in the pathogenesis of CF have recently been gathered [6-8].

Corticosteroids and ibuprofen have been proven to slow down the impairment of the pulmonary function in CF patients but their use is limited by the frequency and severity of adverse effects [9-12].

Indeed, CF patients are often treated with corticosteroids during pulmonary infective breakthroughs, as shown by Oermann who reported that 41% of physicians in CF centres prescribe long-lasting oral steroids [13]. Pharmacological activity of corticosteroids varies from one patient to another depending on drug absorption especially if administered by aerosol. New systems for slow release of corticosteroids which maintain therapeutic systemic concentrations for a prolonged time using a low quantity of drug, would therefore be useful for the treatment of CF patients. A novel strategy for delivering low doses of steroids for long periods through the infusion of autologous erythrocytes loaded with dexamethasone has been recently set up [14,15]. It has been demonstrated that encapsulated erythrocites present normal morphology, normal metabolic and antigenic properties, and normal survival in vivo [16]. Furthermore a pharmacokinetics study demonstrated that consistent and persistent plasma level can be maintained up to one month after infusion independently of the starting amount of encapsulated dexamethasone 21-P [17].

A recent study suggested the feasibility and a favourable effect of therapy with low doses of corticosteroids delivered through engineered erythrocytes in CF patients up to 15 months of therapy [17]. This study extends the follow up of these patients to 24 months and presents a further analysis of safety and efficacy of this therapy.

## Methods

### Study population

Patients were enrolled at the CF Unit of the Bambino Gesù Hospital, Rome, if they had a definite diagnosis of CF through at least two sweat tests according to Gibson and Cook [18], and through a research of mutation of CFTR gene. Patients entered the study if they had a forced expiratory volume in 1 second (FEV1) <70%, puberty development completed (Tanner V level stage), pancreatic insufficiency (fat balance evaluation <93% and/or faecal elastase <200 μg/g faeces), and chronic bacterial pulmonary infection requiring frequent cycles of intravenous antibiotic therapy. Patients were excluded if they underwent systemic corticosteroid therapy in the three months preceding the experimental treatment or were on therapy with non-steroidal anti-inflammatory drugs (NSADs), if they had liver CF disease, allergic bronchopulmonary aspergillosis, or positive tuberculin test.

The study has been approved by the Ethics Committee of Paediatric Hospital Bambino Gesù and all the patients (or their parents/guardians) have signed an informed consent. Two study groups were considered for this study: individuals who underwent the experimental treatment were compared to a group of patients who followed a standard treatment, who fulfilled the enrolment criteria, and who were matched to the experimental group by gender, age ( ± 2 years), and severity of the disease (FEV1 at enrolment ± 10%; Shwachman index ± 20; Crispin score ± 5) [19,20].

The treatment was not randomised and the assignment was based on the patient's consent. 

### Treatment

The experimental treatment consists in collecting 50 ml of blood into a syringe containing heparin. Erythrocytes are loaded with dexamethasone 21-phosphate through lysis in a hypotonic solution, the drug is entrapped, and the cells are resealed with the use of a "Red Cell Loader" device [14]. Loaded red cells are then reinfused into the original patient resulting in released quantities of dexamethasone 21-phosphate ranging from 0.1 to 0.2 nmol/ml of plasma for up to one month [17]. The entire procedure requires two hours to be completed. Patients in the experimental group received the treatment once a month for a period of 24 months.

All patients followed standard prevention therapies, treatment of chronic pulmonary infection and pancreatic insufficiency (aerosol therapy with DNase and beclomethasone, Pulmozyme, daily FKT, vitamins, pancreatic enzymes), and received antibiotic treatment in case of infective relapses.

### Follow up and outcome

All patients underwent Bone Mineralometry (BM), ocular tonometry, and arterial pressure measurement before entering the trial. Clinical, bacterial, and functional respiratory data were monitored for 12 months before and in the 24 months after starting the experimental treatment, and for a corresponding period of time (36 months) in patients assigned to standard treatment. Patients were continuously monitored for the inflammatory (c-reactive protein, erythrosedimentation rate), infective (white blood cell count, culture of expectoration with antibiogram), and nutritional parameters (weight), and underwent functional pulmonary tests (FEV1 and forced vital capacity (FVC) at any control. All patients were invited to be visited once a month. As for safety of treatment, we studied the oral glucose tolerance test (every year), glycated haemoglobin (every month), bone mineral density, and ocular tonometry (at the end of the study), while blood pressure, skin lesions, and other side effects were monitored at each visit. We have analysed Shwachman and Crispin indexes at the end of treatment, and FEV1 trend over time. Episodes of infective bronchopulmonary breakthrough according to Ramsey criteria were also considered in the outcome measures [21] as well as the number of visits performed during the study period.

### Statistical analysis

Since this study was designed as a pilot trial we did not perform any estimation of the sample size required for showing any effect of the treatment or any difference in the rate of side effects.

Variations in the Shwachman and Crispin indexes were evaluated through the Student t test. We have used the standard deviations (Z scores) of FEV1 for each visit based on the patient average of measures during the 12 months preceding the trial. The mean and standard deviation of FEV1 Z scores were calculated for each time interval. A general linear model repeated-measures ANOVA was used to compare

FEV1 Z scores in patients with the experimental treatment with those with standard therapy. Linear regression was used to describe the FEV1 Z score trend over time. The incidence density rates of infectious episodes have been compared through the Student t test, whereas frequencies of side effects in the two groups have been compared through the Chi square test. Analyses were performed with the statistical package SPSS (version 11.5, SPSS, Inc).

## Results

We enrolled 17 patients in the experimental group and nine patients in the standard treatment group. Of the 17 patients in the experimental group, eight received less than four administrations of dexamethasone phosphate through loaded erythrocytes and therefore were excluded from further analyses. These patients (2 males, 6 females; median age 23,5; SD:7 years) had similar demographic characteristics of patients continuing the experimental therapy and did not continue the red cell loading because of personal discomfort in receiving the treatment, or for difficulties in being regularly visited and treated by our centre. None of these patients developed any side effects.

The baseline characteristics of patients are illustrated in Table 1. The two groups showed similar demographic and clinical characteristics at the beginning of the trial.

**Table 1:** Baseline characteristics of the patients enrolled in the trial

	**Erythrocyte mediated steroid therapy**	**Ordinary therapy**
No. of patients	9	9
Females, no.	5	5
Age at enrolment, years (range)	19.8 (15–26)	20.0 (13–26)
Colonization by P. aeruginosa	6	7
Colonization by *B. cepacia*	3	1
Colonization by *Staphilococcus aureus*	-	1

Two patients in the experimental treatment group, and two in the standard treatment group had a diagnosis of diabetes type 1 before entering the study. Six patients in the experimental treatment group and nine in the standard treatment group received TOBI therapy during the study.

None of the patients in the experimental treatment group developed diabetes, while two patients in the standard treatment group did (22%). We did not observe any decrease of bone mineral density, or any increase of ocular pressure in the two groups of patients. No patients developed cataract, arterial hypertension, or cutaneous striae rubrae, and no death was observed.

Shwachman and Crispin scores did not vary in the two groups during follow up, as indicated in Table 2. Average FEV1 at enrolment, at 12, and 24 months are reported in Table 3. Despite we did not observe any significant difference in the average FEV1 due to the small sample size, these measures indicate an opposite trend in the two groups: FEV1 tended to increase in the experimental treatment group, while in the standard treatment a decreasing trend was observed.

**Table 2:** Variation of Shwachman and Crispin indexes during follow up

	**Erythrocyte mediated steroid therapy**	**Ordinary therapy**	**P value**
Mean Shwachman index at enrolment (95% CI)	71.3 (36.2–106.4)	70.9 (41.3–100.5)	0.73
Mean Shwachman index at 12 months of follow up (95% CI)	62.6 (20.0–103.2)	68.4 (39.2–97.6)	0.68
Mean Shwachman index at 24 months of follow up (95% CI)	66.8 (21.9–111.7)	68.0 (37.6–98.4)	0.83
Mean Crispin index at enrollment (95% CI)	13.7 (4.5–22.6)	12.7 (5.3–20.1)	0.76
Mean Crispin index at 24 months of follow up (95% CI)	13.6 (5.2–22.0)	12.9 (5.9–19.9)	0.48

**Table 3:** Variation of FEV1 during follow up

	**Erythrocyte mediated steroid therapy**	**Ordinary therapy**	**P value**
FEV1 at enrolment (-12 months), average (95% CI)	53.8 (25.6–82.0)	56.8 (30.0–83.6)	0.68
FEV1 at time +12, average (95% CI)	56.7 (23.8–89.6)	55.1 (26.5–83.7)	0.83
FEV1 at time +24, average (95% CI)	59.8 (22.4–97.2)	53.2 (21.7–84.7)	0.48

**Figure 1:**
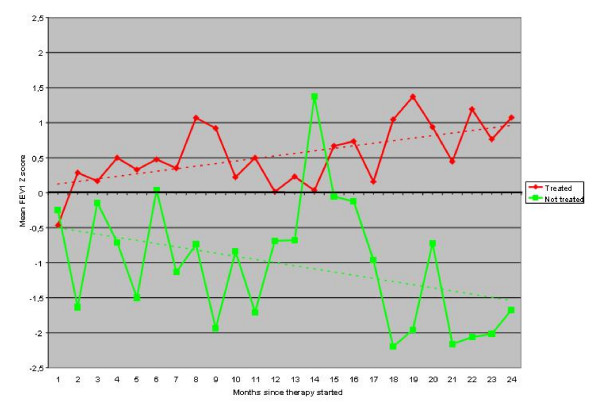
Comparison of FEV1 z-scores in treated and untreatedpatients over time. Continuous lines represent the average z-score ofFEV1 by month. Dotted lines represent the interpolation of the z-scores by linear regression. Comparison of the two groups through general linear model repeated-measures. ANOVA yielded a P value = 0.04.

The analysis of FEV1 Z scores over time (Figure 1) confirmed opposite trends in the two groups. In the experimental treatment group we observed a progressive improvement of FEV1 compared to the average FEV1 observed before starting experimental therapy. At the end of the follow up period, FEV1 in the experimental treatment group was nearly one standard deviation higher than the reference period. On the contrary, in patients with standard treatment we observed a progressive decrease of FEV1 with a value at 24 months 1.5 standard deviations less than the reference period. The differences observed were statistically significant (P = 0.04).

Patients in the experimental treatment group underwent a number of visits higher than the standard treatment group during follow up (experimental treatment: 1.1 visit per person month; standard treatment: 0.7 visit per person month; P < 0.01). The incidence of infectious episodes was slightly higher in patients who underwent the experimental treatment (experimental treatment: 0.24 per person month; standard: 0.18 per person month; P=0.08).

None of the eight patients originally enrolled in the experimental therapy group who did not complete the study died or underwent transplantation, but all of them had a FEV1 stable or lower than measured at the beginning of the trial compared to that measured after 24 months.

## Discussion

This study provides further elements supporting that therapy with dexamethasone 21-phosphate-delivered in autologous erythrocytes may be safe and moderately efficacious in CF patients.

An earlier study performed on the same patients focused on pharmacokinetics and technical details of therapy with dexamethasone 21-phosphate-delivered in autologous erythrocytes [17]. In the present manuscript we reviewed FEV1 values over time in the same patients, and prolonged the follow up period. The present results substantially reduce the apparent effect of treatment on functional respiratory tests previously reported. However, the benefits associated with this therapy seem to be extended beyond 15 months of follow up.

The availability of a safe means to administer corticosteroids may be advantageous for CF patients, as well as for other diseases requiring chronic use of these drugs.

Studies performed in North America on CF patients indicated that treatment with oral steroids was efficacious in improving respiratory function but it caused several adverse effects, such as a significant modification of glucose metabolism and growth failure [10,11], particularly after two years of treatment. In fact, the low doses of steroids administered with loaded erythrocytes may reduce these side effects. All patients included in this study completed sexual development and therefore we were not able to measure any effect on growth. However, during the follow up period no patients in the experimental group developed diabetes, cataract, or hypertension or other typical side effects of steroid treatment such as Cushingoid aspect or skin manifestations.

The main result of this study is a constant improvement of FEV1 in patients undergoing the experimental treatment compared to a gradual decrease of the same parameter in the standard therapy group. The average clinic and radiologic indexes did not vary in the two groups since such parameters are certainly less sensitive in defining the patient's pulmonary performance in the short term.

Although the study was not randomized, the patients of the two group at the beginning of the trial were similar for age, gender, colonization, respiratory function, and disease stage. We did not perform an intention to treat analysis given the exploratory nature of this pilot study. Inclusion of patients originally assigned to the experimental treatment would have resulted, however, in a much smaller difference in FEV1 over time between the two groups. The number of infective relapses that have required antibiotic intravenous therapy, differently from the previous study, was similar in the two groups, although the average number of episodes was slightly higher in the experimental therapy group. The absence of blinding may be theoretically associated with a more strict control of patients in the experimental group and with an early recognition of infective relapses and, therefore, with a timely treatment. The number of visits per month in the experimental group was actually higher than in the standard treatment group.

Though the experimental therapy included in the study may be useful for controlling chronic inflammation of the airway in CF patients, additional preventive strategies should be developed.

## Conclusion

Although the number of enrolled patients in our study was too low to draw definite conclusions, this new administration technique seems safe and moderately efficacious despite the very low steroid circulating dose. The results obtained by our study cannot be considered definitive but may help planning an experimental, controlled, randomised study which may give more robust results, and may involve patients of different age groups with different doses and administration schemes.

Based on means and standard deviations of FEV1 observed at 24 months in this study, a sample size of 150 patients per group would be sufficient for demonstrating such a difference with a 95% confidence interval and a power of 90%. If a clinical trial with a sufficient number of participants will confirm these preliminary results, an important therapeutic tool will be available to improve the prognosis of CF patients. Moreover studies on corticosteroid drugs in CF paediatric patients need at least five years to assess the control of inflammation in the long term and the occurrence of late side effects. Alternative strategies for inflammation control should focus on the immune imbalance which has been found even in young clinically stable CF patients [22].

## Competing interests

The author(s) declare that they have no competing interests.

## Authors' contributions

VL, SB, and AT recruited the patients and administered the experimental treatment. AET set the methods for the clinical trial and the analysis of data. All authors contributed to prepare the manuscript.

## Pre-publication history

The pre-publication history for this paper can be accessed here:


